# Supplementary material for: Spiking neurons with spatiotemporal dynamics and gain modulation for monolithically integrated memristive neural networks
Source: Nat Commun. 2020 Jul 7;11:3399. doi: 10.1038/s41467-020-17215-3 (PMC7341810; doi:10.1038/s41467-020-17215-3)
Supplement: Supplementary file 1 — Supplementary Information [file 41467_2020_17215_MOESM1_ESM.pdf]

**Supplementary Information**

# Spiking Neurons with Spatiotemporal Dynamics and Gain Modulation for Monolithically Integrated Memristive Neural Networks

**Duan et al.**

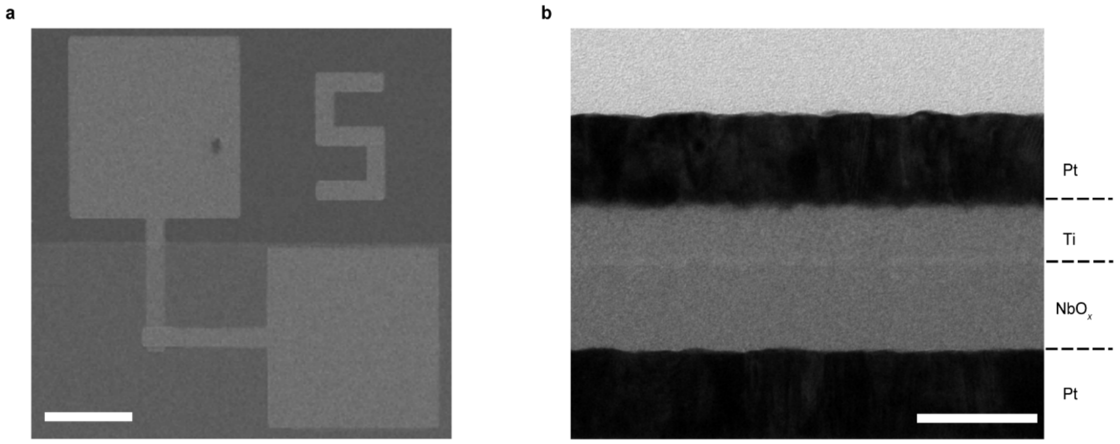


**Supplementary Figure 1. Structure of the $NbO_x$ threshold switching device.** (**a**) SEM image. Scale bar, 20 µm. (**b**) Cross-sectional TEM image of a $NbO_x$ device. Scale bar, 50 nm.

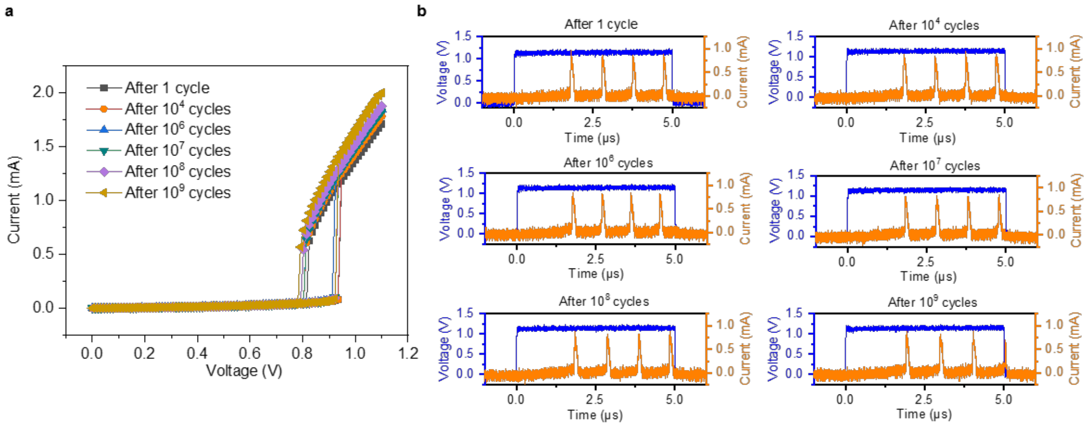


**Supplementary Figure 2. Endurance of Pt/Ti/$NbO_x$/Pt/Ti threshold switching device.** (**a**) *I*-*V* characteristics of Pt/Ti/$NbO_x$/Pt/Ti device after 1, $10^4$, $10^6$, $10^7$, $10^8$ and $10^9$ switching cycles. (**b**) The firing behavior of Pt/Ti/$NbO_x$/Pt/Ti device after 1, $10^4$, $10^6$, $10^7$, $10^8$ and $10^9$ switching cycles.

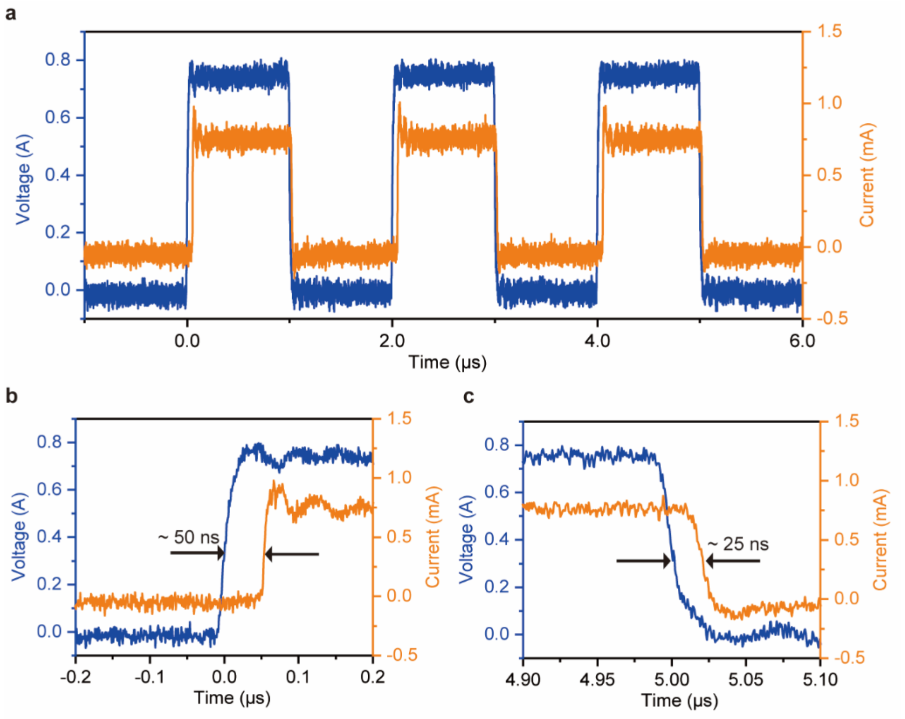


**Supplementary Figure 3. Transient switching response of the $NbO_x$ threshold switching device.** (**a**) Current waveform (orange curve) of the $NbO_x$ device upon application of the voltage waveform (blue curve). (**b**) The switching speed is <50 ns from off- to on-state. (**c**) The switching speed is <25 ns from on- to off-state.

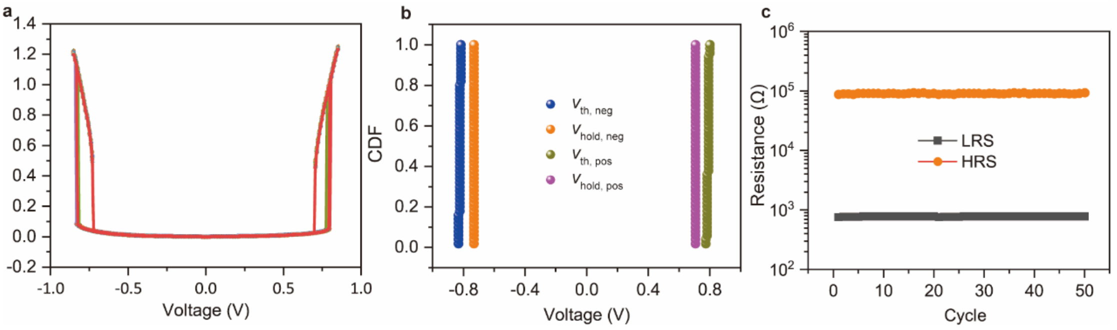


**Supplementary Figure 4. Cycle-to-cycle variation of the $NbO_x$ threshold switching device**. (**a**) *I*-*V* characteristics of the device in 50 repeated cycles. (**b**) Cumulative plots of $V_{th,\ pos}$, $V_{hold,\ pos}$, $V_{th,\ neg}$, $V_{hold,\ neg}$. (**c**) Distributions of high and low resistance states of the $NbO_x$ device in 50 repeated cycles.

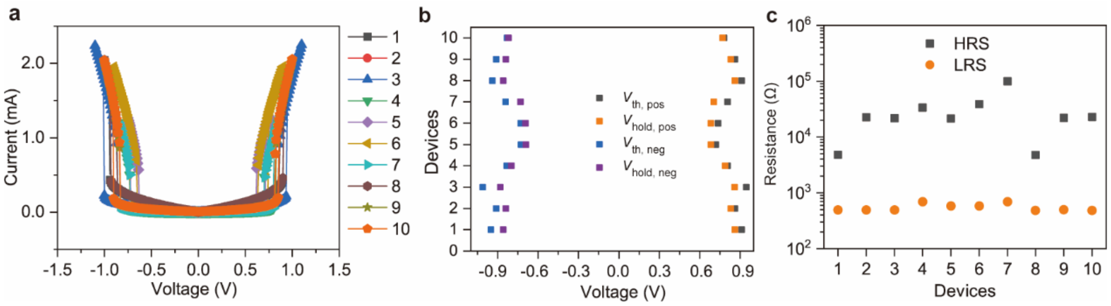


**Supplementary Figure 5. Device-to-device variation of the $NbO_x$ threshold switching device**. (**a**) *I*-*V* characteristics of the device measured in 10 different Pt/Ti/$NbO_x$/Pt/Ti devices. (**b**) Distributions of $V_{th, pos}$, $V_{hold, pos}$, $V_{th, neg}$, $V_{hold, neg}$ in 10 $NbO_x$ devices. (**c**) Distributions of high and low resistance states in 10 $NbO_x$ devices.

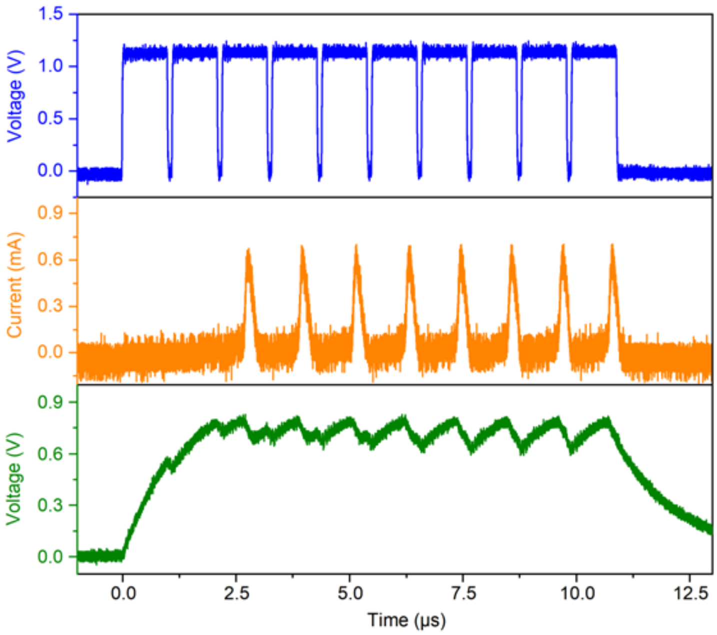


**Supplementary Figure 6.** The input voltage (blue curve), output current (orange curve) and the voltage across the threshold switching device (green curve) using the circuit in Fig. 2d.

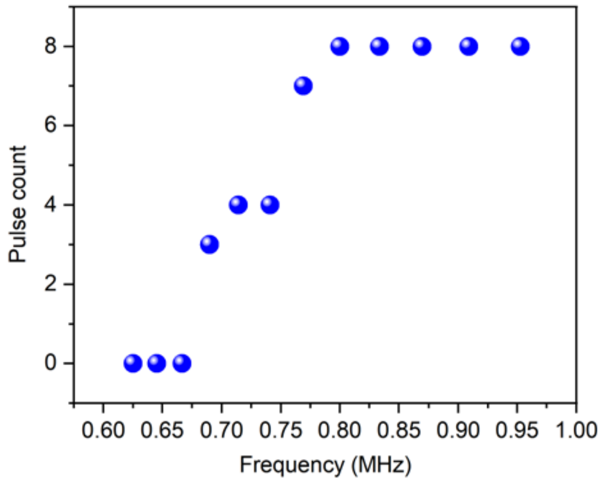


**Supplementary Figure 7.** A pulse count vs. input frequency plot of the artificial neuron (with $R_L$ of 3.6 kΩ, $C_m$ of 30 pF, pulse width of 1 μs, and pulse amplitude of 1.2 V).

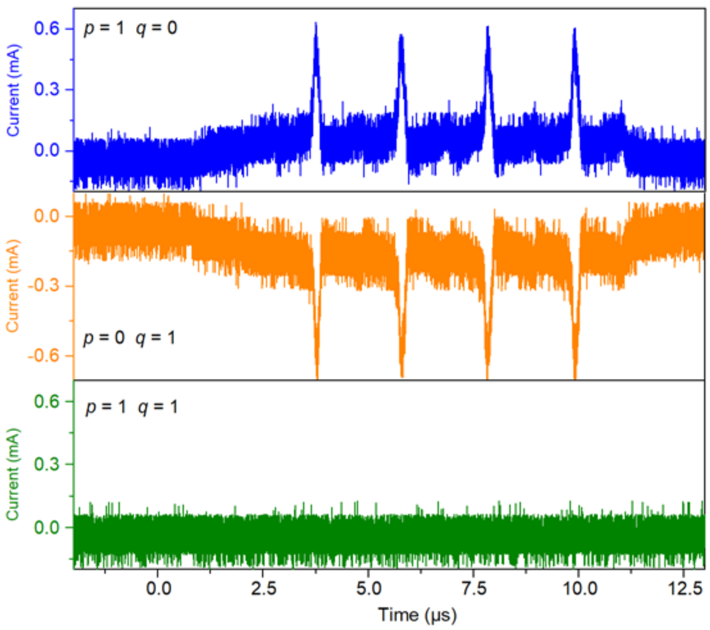


**Supplementary Figure 8. XOR logic implemented using the $NbO_x$ neuron.** 0 V is defined as logic "0" for the inputs, and the logic "1" for p and q are defined to be 0.9 V and –0.9 V, respectively. Output of the neuron when "$p$ = 1, $q$ = 0" (top panel), "$p$ = 0, $q$ = 1" (middle panel), and "$p$ = 1, $q$ = 1" (bottom panel) demonstrates a XOR function.

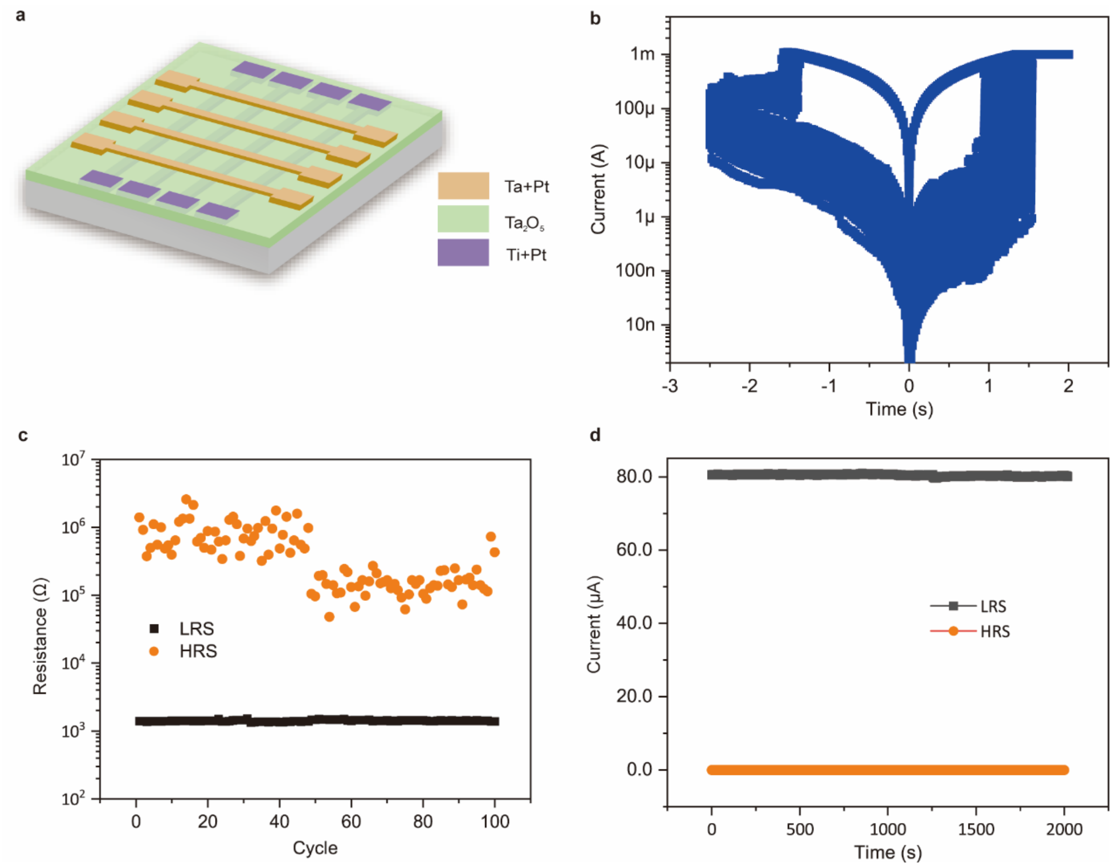


**Supplementary Figure 9. Electrical characteristics of $TaO_x$ synapses.** (**a**) Schematic of the device. (**b**) *I*-*V* characteristics of the Pt/Ta/$Ta_2O_5$/Pt/Ti device repeated for 100 cycles. (**c**) Cycle-to-cycle variation of the Pt/Ta/$Ta_2O_5$/Pt/Ti device. (**d**) Retention of the Pt/Ta/$Ta_2O_5$/Pt/Ti device.

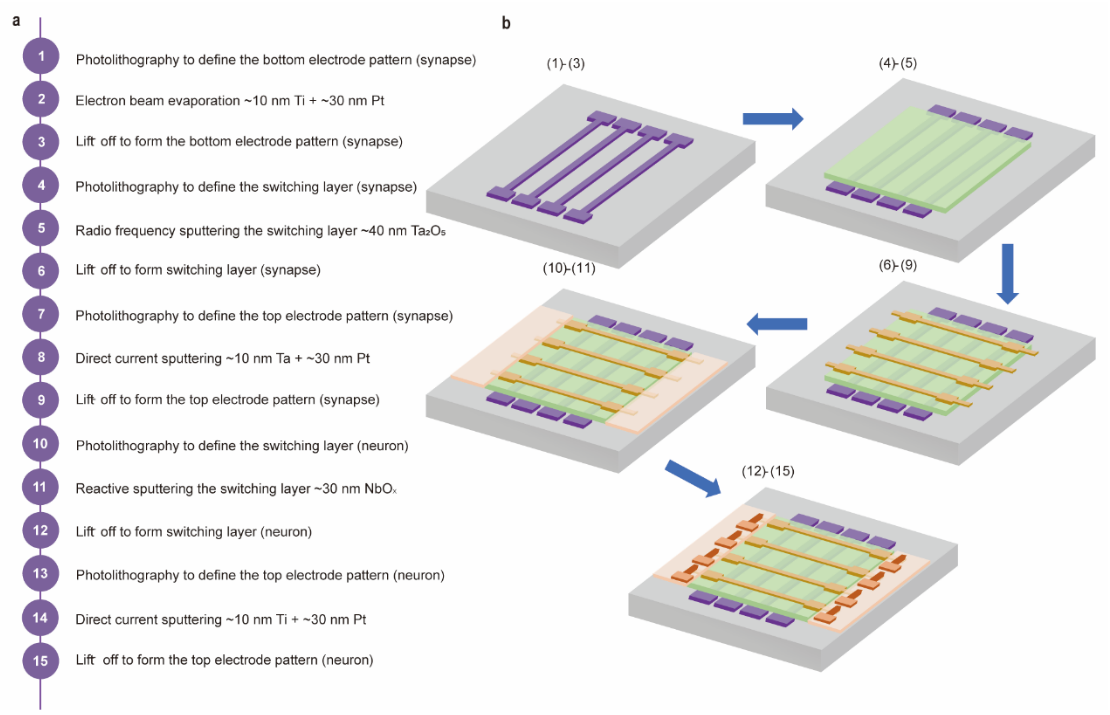


**Supplementary Figure 10. Fabrication process of the fully memristive neural network.** (**a**) The fabrication process of a fully memristive network. (**b**) Schematic diagram of the fabrication.

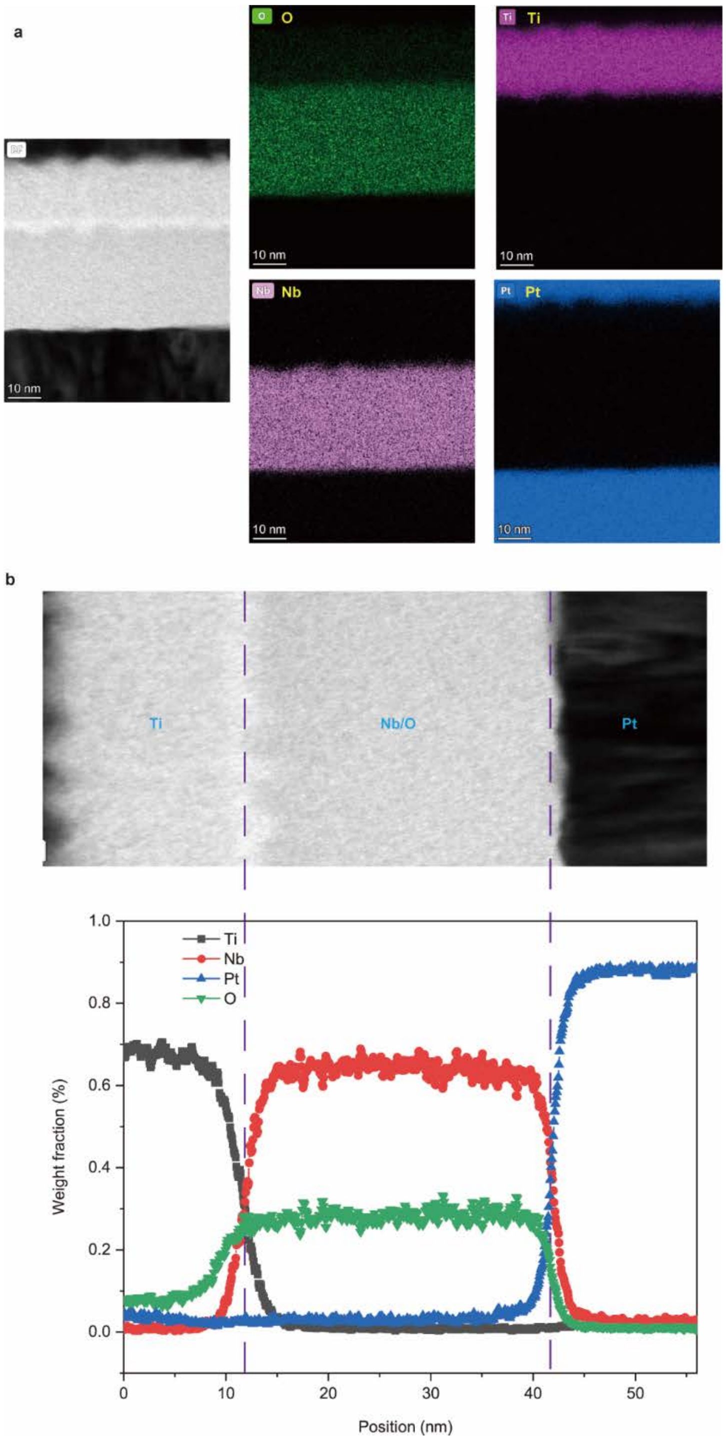


**Supplementary Figure 11. Microstructural and compositional characterization of $NbO_x$ device.** (**a**) Cross-sectional STEM image and corresponding EDS mapping of O, Ti, Nb and Pt elements in the device. (**b**) EDS elemental line profile in the region of the device shown by the STEM image.

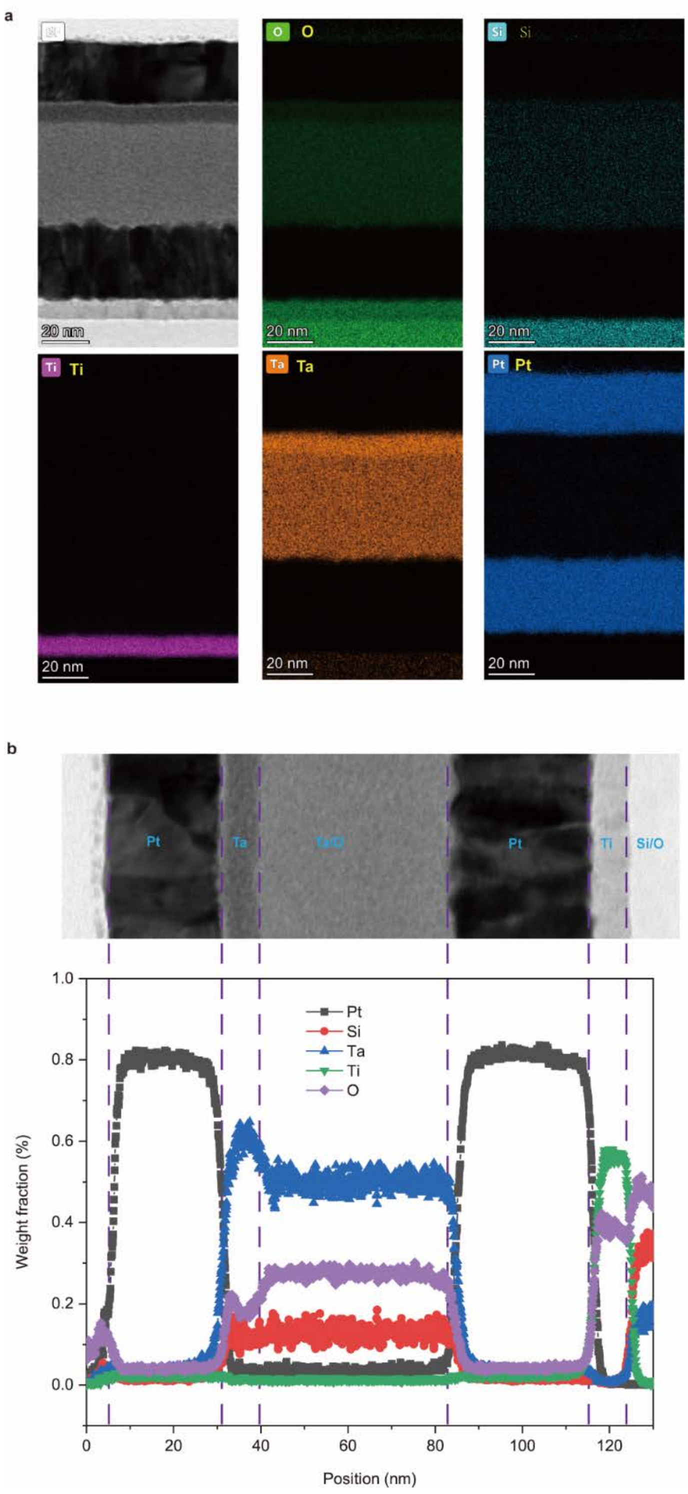


**Supplementary Figure 12. Microstructural and compositional characterization of $TaO_x$ synapses.**

(**a**) Cross-sectional STEM image and corresponding EDS mapping of O, Si, Ti, Ta and Pt elements in the $TaO_x$ device. (**b**) EDS elemental line profile in the region of the device shown by the STEM image.

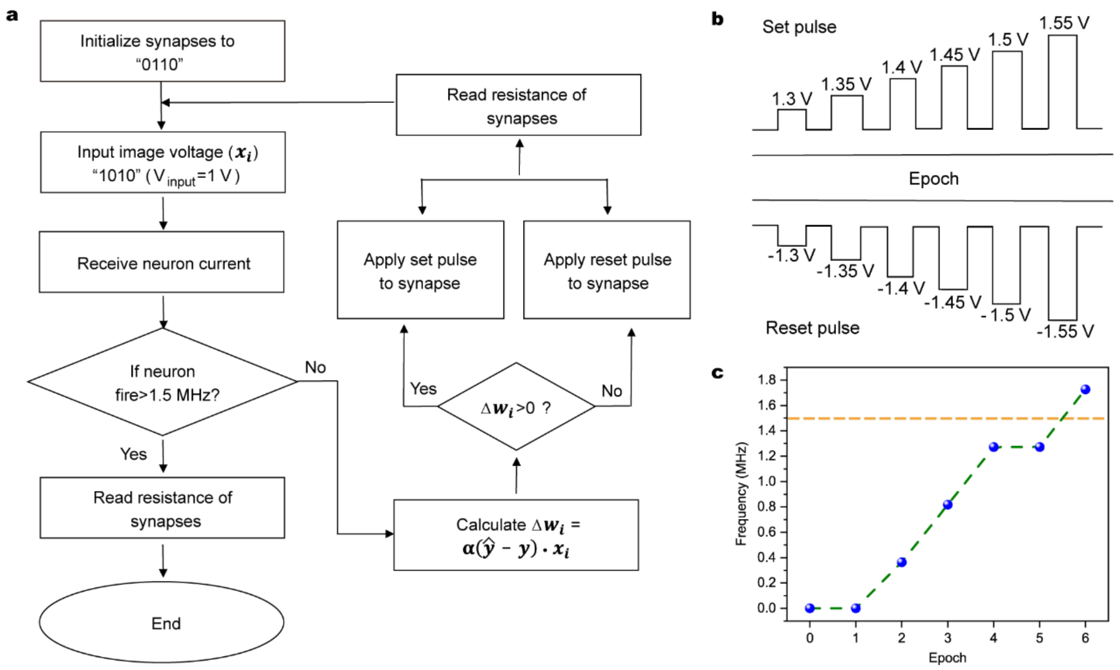


**Supplementary Figure 13. Supervised learning in fully memristive neural networks.** (**a**) Flow chart of the training process. (**b**) Stepped pulses used to modify the synaptic weights during training. (**c**) Evolution of the neuron firing rate with the number of training cycle. The firing rate of the neuron after the 6th epoch exceeds the pre-determined threshold for ending the training.

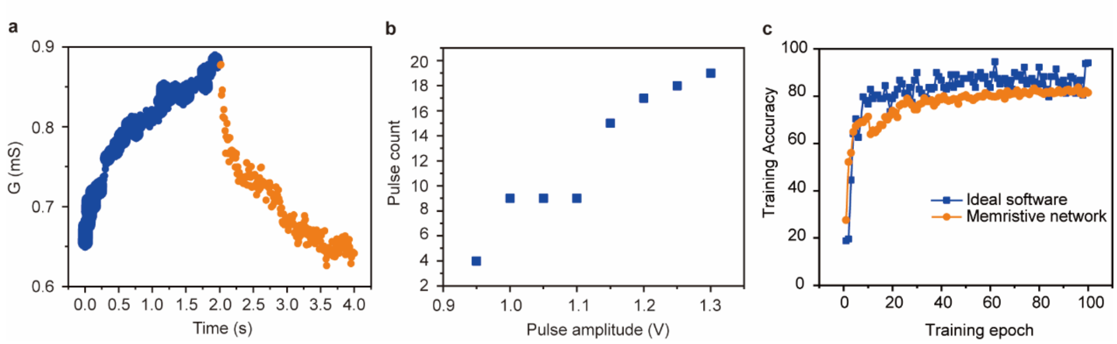


**Supplementary Figure 14. Simulation of large-scale fully memristive spiking neural networks.** (**a**) Continuous conductance tuning of the $TaO_x$ synapses using a pulse train during potentiation (1.1 V, 1 μs) and depression (-0.95 V, 1 μs). (**b**) Neuron firing pulse count as a function of input pulse amplitude (1 μs in width, 0.1 μs in interval with 10 pulse cycles). Neuron is in series with $R_L$ of 2.2 kΩ. (**c**) Evolution of the training accuracy with training epoch. The line with orange circles is the training accuracy of simulated SNN, which reaches 81.45% after 100 epochs. The line with blue squares is the accuracy of software network trained with the same conditions.

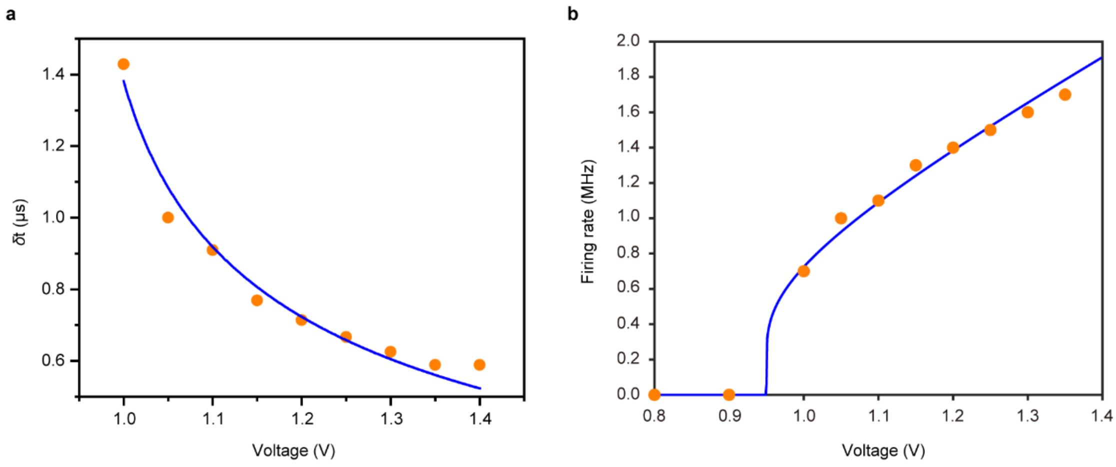


**Supplementary Figure 15. Simulation of coincidence detection.** (**a**) The time ($\delta$t) to obtain each spike firing under different input voltages (orange data point) and the fitting curve (blue curve). (**b**) The firing frequency of neurons under input voltages (orange data point) and the fitting curve (blue curve).

| Figure No. | $R_1$ | $R_2$ | $C_m$ |
|---|---|---|---|
| Fig. 3c – 3d | 3 kΩ | 3 kΩ | 30 pF |
| Fig. 3g – 3h | 3 kΩ | 3 kΩ | 30 pF |
| Fig. 3k – 3l | 2.2 kΩ | 10 kΩ | 30 pF |
| Fig. 4c – 4d | 2.2 kΩ | 2.2 kΩ | 30 pF |

**Supplementary Table 1. Circuit parameters for implementing artificial neurons.**

| **Material** | **Speed** | **Power consumption** |
|---|---|---|
| Ag/Ag:$SiO_xN_y$, Ag/$HfO_2$, Ag/$SiO_2$ diffusive memristors | <500 ns[1-4] | 1.2–480 μW[5-7] |
| $NbO_x$ | <10 ns[8-11] | 10–1600 μW[12-14] |
| $VO_x$ | <700 ps[15-18] | 23.75–2400 μW[19-21] |
| $Ge_2Sb_2Te_5$ | <20 ns[22-24] | ~4.3 μW[24] |
| $NbO_x$ (This work) | < 50/25 ns | ~392 μW |

**Supplementary Table 2. Comparison of switching speed and power consumption for artificial neurons based on different materials and approaches.**

**Supplementary Note 1: Structural and electrical characterization of $NbO_x$ devices**

To characterize the $NbO_x$ device, scanning electron microscopy (SEM) characterization and transmission electron microscopy (TEM) have been carried out and the results are shown in Supplementary Figure 1. It can be seen from Supplementary Figure 1a that the typical dimension of the device is ~$5 \times 5$ μm$^2$. Supplementary Figure 1b further shows a HRTEM image, where the stacking structure can be clearly observed.

We have performed experiments to measure the endurance of the device, and the results show that the device can still function correctly after >$10^9$ switching cycles, as shown in Supplementary Figure 2. Supplementary Figure 3a shows the transient switching response of the $NbO_x$ threshold switching device. It can be found that the switching speed of $NbO_x$ threshold switching device in the present work is <50 ns from off- to on-state (Supplementary Figure 3b) and <25 ns from on- to off-state (Supplementary Figure 3c).

We also examined the cycle-to-cycle and device-to-device variation of the devices. Supplementary Figure 4a shows the *I*-*V* characteristics of the $NbO_x$ device in 50 repeated cycles, showing excellent cycle-to-cycle uniformity. The cycle-to-cycle fluctuations in $V_{\text{th, pos}}$, $V_{\text{hold, pos}}$, $V_{\text{th, neg}}$, $V_{\text{hold, neg}}$ as well as high and low resistance states are further plotted in Supplementary Figure 4b,c, which once again demonstrates very low cycle-to-cycle variation.

Supplementary Figure 5a–c exhibits *I*-*V* characteristics measured in 10 different

Pt/Ti/$NbO_x$/Pt/Ti devices and the device-to-device distributions of $V_{th, pos}$, $V_{hold, pos}$, $V_{th, neg}$, $V_{hold, neg}$ as well as high and low resistance states, showing relatively low device-to-device variations. These results in Supplementary Figures 4 and 5 demonstrate that the Pt/Ti/$NbO_x$/Pt/Ti devices have acceptable cycle-to-cycle and device-to-device variations, making them qualified for building artificial neurons.

**Supplementary Note 2: spike count vs. input conditions in spiking neuron**

Supplementary Figure 6 shows the input voltage, current response and the voltage across the threshold switching device as functions of time, which illustrate the dynamics during threshold switching of $NbO_x$.

Supplementary Figure 7 exhibits a plot of spike count vs. input frequency (with $R_L$ of 3.6 kΩ, $C_m$ of 30 pF, pulse width of 1 μs, and pulse amplitude of 1.2 V), where the pulse count increases as the input frequency increases. The plot is qualitatively consistent with the case without modulatory input ($m$ = 0) in Fig. 4d.

**Supplementary Note 3: The 3D surface equation of Fig. 3d, h**

We have fitted the experimental results in Fig. 3d, h and found the data can be approximated by the following empirical equations.

For Fig. 3d:

$$f(V_1,V_2) = b_1 e^{\beta_1 (V_1 - 0.6)} + b_2 e^{\beta_2 (V_2 - 0.6)} + b_3 (V_1 - 0.6)(V_2 - 0.6) + b_4 \quad (1)$$

where $b_1 = 37.7$, $b_2 = 47.41$, $b_3 = -221.3$, $b_4 = -85.11$, $\beta_1 = 1.335$, $\beta_2 = 1.09$

and for Fig. 3h:

$$f(\Delta t_1, \Delta t_2) = c_1 e^{-\lambda_1 \Delta t_1} + c_2 e^{-\lambda_2 \Delta t_2} + c_3 \Delta t_1 \Delta t_2 + c_4 \quad (2)$$

where $c_1 = 15.11$, $c_2 = 10.47$, $c_3 = 45.74$, $c_4 = -10.58$, $\lambda_1 = 1.694$, $\lambda_2 = 1.737$

**Supplementary Note 4: Electrical characterization of the $TaO_x$ synapse device**

Supplementary Figure 9a schematically illustrates the crossbar structure of $Pt/Ta/Ta_2O_5/Pt/Ti$ synapse devices fabricated on $SiO_2/Si$ substrates. Supplementary Figure 9b shows typical resistive switching characteristics of a $Pt/Ta/Ta_2O_5/Pt/Ti$ device with a current compliance of 1mA. Reproducible resistive switching can be achieved by applying a set voltage of >1.5 V and a reset voltage of < –2.5 V with 100 cycles, showing typical bipolar resistive switching. Supplementary Figure 9c further shows that the distributions of HRS and LRS within different *I-V* cycles, where cycle-to-cycle variation mainly exists in off state. Meanwhile, Supplementary Figure 9d shows that the HRS and LRS of the $Pt/Ta/Ta_2O_5/Pt/Ti$ device remain stable for >2000 s without noticeable degradation, indicating a nonvolatile nature.

**Supplementary Note 5: Fabrication of the fully memristive neural networks**

The fabrication process of the fully memristive neural network is schematically shown in Supplementary Figure 10a, which was fabricated on $SiO_2$/Si substrates. First, 30 nm thick Pt film with 5 nm thick Ti adhesion layer of 2 μm width was deposited by e-beam evaporation, where the patterning of the bottom electrodes was done by photo lithography and lift-off processes. Afterwards, photo lithography was used to form the patterns of the switching layer, followed by RF sputtering and lift-off processes. The switching layer of synaptic devices in this study was $Ta_2O_5$ (30 nm). Subsequently, a Ta (10 nm)/Pt (30 nm) of 2 μm width as a middle electrode (ME) was formed to cover the BE vertically, where the patterning of the middle electrodes was done by photo lithography and lift-off processes, which resulted in Pt/Ta/$Ta_2O_5$/Pt/Ti synaptic devices. Next, photo lithography was performed to define the switching layer of the artificial neurons, and $NbO_x$ film (30 nm) was deposited as the switching layer by DC reactive sputtering using Ar and $O_2$ mixture gases (Ar: 10 mTorr, $O_2$: 70 mTorr). Finally, 10 nm thick Ti was deposited as the top electrode and capped by 30 nm thick Pt protection layer by DC sputtering, where the patterning of the top electrodes was done by photo lithography and lift-off processes. A schematic configuration of the cell structure is depicted in Supplementary Figure 10b. Supplementary Figure 11a shows energy dispersive spectrometer (EDS) elemental mapping of O, Ti, Nb and Pt of a $NbO_x$ device, and Supplementary Figure 11b further shows the line-scan results. The results of EDS mapping and line-scan of the $TaO_x$ devices are shown in Supplementary Figure 12a and Supplementary Figure 12b.

**Supplementary Note 6: Supervised learning in fully memristive neural networks**

Supplementary Figure 13a shows the training process flow chart of achieving supervised learning in fully memristive neural networks. We used a simplified *δ-rule* rule to modify the synaptic weights. The role of artificial neurons is to produce an output ($y$) for an input vector $x = (x_1, x_2, \cdots\cdots x_n)$. In order for the neuron to achieve the desired output, we need to train it. The training samples are a series of known $x$ and $\hat{y}$, where $\hat{y}$ represents the expected correct output. The following equation is used to describe the error between the actual output and the expected correct output:

$$E = \frac{1}{2}(y - \hat{y})^2 \quad (3)$$

In order to achieve desired output of the neuron, every time when the weight $w_i$ is modified, the following update value applies $\Delta w_i = -\alpha \frac{\partial E}{\partial w_i}$, where $\alpha$ is a factor that indicates how fast the learning speed is, usually called the learning rate.

$$\frac{\partial E}{\partial w_i} = (y - \hat{y}) \times \sigma'(s) \times x_i \quad (4)$$

The value of $\frac{\partial E}{\partial w_i}$ is defined in Eq. (4). Since $\sigma'(s)$ >0 it does not affect the direction of the weight correction, so it can be ignored, so $\Delta w_i = \alpha(y - \hat{y}) \times x_i$, which is the *δ-rule*.

In our experiment, we simplified the *δ-rule* by defining $y$ = 1 when the firing frequency of the neuron is >1.5 MHz, $y$ is the actual neuron output. If neuron firing frequency is < 1.5 MHz, $y$ = 0. During training, set/reset pulses were applied to modify

the weight by judging whether $\Delta w_i$ >0.

The network structure is shown in Fig. 5h. Initially, the synapses $S_2$ and $S_3$ were in the high conductance state, while the $S_1$ and $S_4$ were in low conductance state, that is "0110". Meanwhile, the input is "1010", that is a train of rectangular spikes were applied to the first and third row of synapses, the second and fourth row was kept at zero bias, i.e. $(x_1 = 1, x_2 = 0, x_3 = 1, x_4 = 0)$. The input image pattern "1010" is 1 V in amplitude, 1 μs in width, 0.1 μs in interval and repeated for 10 cycles at the beginning of each training cycle. When the input pattern "1010" was applied, we monitore the neuron current to determine if the neuron fires. If the firing frequency of neuron is > 1.5 MHz, the learning is ended, since the neural network recognizes "1010" by training. If the firing frequency of neuron is <1.5 MHz, we will judge whether $\Delta w_i$ >0. If $\Delta w_i$ >0, set pulse will be applied to the synapses. The specific pulse voltage applied to change the synaptic weight is shown in Supplementary Figure 13b. If $\Delta w_i \leq 0$, reset pulses will be applied to the synapses. The specific pulse voltage applied to change the synaptic weight is shown in Supplementary Figure 13b. By applying a stepped voltage, the network will learn the desired image gradually. Supplementary Figure 13c shows the results of the neuron firing for every training cycle. The firing frequency of neuron after the 6th epoch exceeds the pre-determined value.

## Supplementary Note 7: Simulation of large-scale fully memristive spiking neural networks

A 3-layer spiking neural network (SNN) was built in Python based on experimental results. The simulated SNN was composed of 784 input neurons, 100 hidden neurons and 10 output neurons, where the 784 inputs and 10 outputs corresponded to a MNIST data size of 28×28 and 10 possible classes (from 0 to 9), respectively. To model the modulation of conductance of $TaO_x$ synapse under pulse train condition, the increase and decrease of conductance during set operation (1.1 V, 1 μs) and reset operation (–0.95 V, 1 μs) were measured (shown in Supplementary Figure 14a) and used for the simulation. During the simulation, we tuned the device conductance by write-and-verify strategy, and conductance discreteness and noise were taken in to consideration. Both in the forward and backward passes, the device conductance was clamped within the range of (–300 μS, 300 μS) and quantized to 7 bit.

The $NbO_x$ device was used as LIF neuron (as shown in Fig. 2), where the parameters were extracted from experimental data (as shown in Fig. 2b, 2e, 2f). The main parameters of the LIF neuron used in the simulation are high resistance state (5 kΩ), series resistance (10 Ω), parallel capacitance (3 μF) and state update time step (1 μs).

The neural network was trained online with Backpropagation (BP) algorithm. The images were first converted into Poisson spike trains and input to the memristor array, and then the $NbO_x$ neurons in the hidden layer propagate the spikes to the next array, at last the output neurons spikes and the index of the most frequently spiking neuron was taken as prediction result. In the backward process, since the input-output function of a

LIF neuron is step function, which has infinite gradient, we replaced it with a soft spike function such as sigmoid function to get the gradient.

**Supplementary Note 8: Simulation of large-scale coincidence detection**

In order to realize the task of coincidence detection, a simulation was performed based on Brian2 simulator. The parameters of the neuron were extracted from electrical experiments, as shown in Supplementary Figure 15. We used the following neuron model, where the membrane potential $V$(t) is governed by:

$$\tau_m \frac{dV(t)}{dt} = -(V(t) - V_0) \qquad (5)$$

Furthermore, according to the experimental data in Supplementary Figure 15b, the time ($\delta$t) for each spike firing is obtained, and integration of Eq. (5) can lead to $\delta$t–$V$ equation, which is described as:

$$\delta t = \tau_m \cdot \ln(\frac{V}{V - V_0}) \qquad (6)$$

where $\tau_m$ = 0.461 μs, $V_0$ = 0.95 V, as shown in Supplementary Figure 15a. $\tau_m$ and $V_0$ can be further introduced into Eq. (5) to get the firing curve of the neuron (Supplementary Figure 15b).

**Supplementary Note 9: Comparison of artificial neurons based on different materials and approaches**

We have compared the speed and power consumption of existing approaches for implementing artificial neurons, including neurons based on diffusive memristors, threshold switching devices including $NbO_x$ and $VO_x$, as well as phase change materials like $Ge_2Sb_2Te_5$, along with our $NbO_x$ devices in the present work (Supplementary Table 2). Here, the power consumption refers to the peak power consumed by the threshold switching (or resistive switching) device when the neuron fires. It can be found that the switching speed of $NbO_x$ threshold switching device in the present work is faster than 50 ns from off- to on-state and faster than 25 ns from on- to off-state (Supplementary Figure 3). The intrinsic switching speed of $NbO_x$ was reported to be <10 ns, so the switching speed of $NbO_x$ based devices is very fast and promising. Previously reported results on the power consumption of $NbO_x$ based neurons have shown significant variation, ranging from 10–1600 μW. It is worthwhile noting that latest studies on $NbO_x$ revealed that the threshold switching effects in $NbO_x$ can be achieved by a trap-assisted conduction mechanism similar to Poole-Frenkel model with moderate Joule heating[25,26], which therefore suggests much lower switching temperature than insulator-metal transition. This actually implies large potential in further optimizing the power consumption of $NbO_x$ based neurons.

**Supplementary References:**


1. Yoo, J. et al. Field-induced nucleation in threshold switching characteristics of electrochemical metallization devices. *Appl. Phys. Lett.* **111**, 063109 (2017).

2. Grisafe, B. et al. Performance Enhancement of Ag/$HfO_2$ Metal Ion Threshold Switch Cross-Point Selectors. *IEEE Electron Device Lett*. **40**, 1602-1605 (2019).

3. Midya, R. et al. Anatomy of Ag/$HfO_2$-based selectors with $10^{10}$ nonlinearity. *Adv. Mater*. **29**, 1604457 (2017).

4. Wang, Z. et al. Memristors with diffusive dynamics as synaptic emulators for neuromorphic computing. *Nat. Mater.* **16**, 101-108 (2017).

5. Wang, Z. et al. Fully memristive neural networks for pattern classification with unsupervised learning. *Nat. Electron.* **1**, 137-145 (2018).

6. Lee, D. et al. Various threshold switching devices for integrate and fire neuron applications. *Adv. Electron. Mater.* **5**, 1800866 (2019)

7. Zhang, X. et al. An artificial neuron based on a threshold switching memristor. *IEEE Electron Device Lett*. **39**, 308-311 (2018).

8. Park, J. et al. $NbO_2$ based threshold switch device with high operating temperature (> 85 °C) for steep-slope MOSFET (~2mV/dec) with ultra-low voltage operation and improved delay time. *2017 IEEE Int. Electron Devices Meet. (IEDM)* **23**, 7. 1-7. 4 (2017).

9. Pickett, M. D. & Williams, R. S. Sub-100 fJ and sub-nanosecond thermally driven threshold switching in niobium oxide crosspoint nanodevices. *Nanotechnology* **23**, 215202 (2012).

10. Luo, Q. et al. $Nb_{1-x}O_2$ based Universal Selector with Ultra-high Endurance (> $10^{12}$), high speed (10 ns) and Excellent $V_{th}$ Stability. *2019 Symposium on VLSI Technology*. T236-T237 (2019).

11. Wang, Z., Kumar, S., Nishi, Y. & Wong, H. S. P. Transient dynamics of $NbO_x$ threshold switches explained by Poole-Frenkel based thermal feedback mechanism. *Appl. Phys. Lett.* **112**, 193503 (2018).

12. Pickett, M. D., Medeiros-Ribeiro, G. & Williams, R. S. A scalable neuristor built with Mott memristors. *Nat. Mater*. **12**, 114-117 (2013).

13. Moon, K. et al. High density neuromorphic system with Mo/$Pr_{0.7}Ca_{0.3}MnO_3$ synapse and $NbO_2$ IMT oscillator neuron. *2017 IEEE Int. Electron Devices Meet. (IEDM)* **17**, 6. 1-6. 4 (2015).

14. Jerry, M. et al. Ultra-low power probabilistic IMT neurons for stochastic sampling machines. *2017 Symposium on VLSI Technology.* T186-T187 (2017)

15. Becker, M. F. et al. Femtosecond laser excitation of the semiconductor-metal phase transition in $VO_2$. *Appl. Phys. Lett.* **65**, 1507-1509 (1994).

16. Cavalleri, A. et al. Femtosecond structural dynamics in $VO_2$ during an ultrafast solid-solid phase transition. *Phys. Rev. Lett*. **87**, 237401 (2001).

17. Jerry, M. et al. Dynamics of electrically driven sub-nanosecond switching in vanadium dioxide. *2016 IEEE Silicon Nanoelectronics Workshop (SNW).* 26-27 (2016)

18. Yi, W. et al. Biological plausibility and stochasticity in scalable $VO_2$ active memristor neurons. *Nat. Commun.* **9**, 1-10 (2018).

19. Lin, J. et al. Low-voltage artificial neuron using feedback engineered insulator-to-metal-transition devices. *2016 IEEE Int. Electron Devices Meet. (IEDM*) **34**, 5.1-5.4 (2016).

20. Shukla, N. et al. Ultra low power coupled oscillator arrays for computer vision applications. *2016 IEEE Symposium on VLSI Technology*. 1-2 (2016)

21. Jerry, M. et al. Phase transition oxide neuron for spiking neural networks. *2016 74th Annual Device Research Conference (DRC)* 1-2 (2016).

22. Loke, D. et al. *Science* **336**, 1566 (2012).

23. Cheng, H. Y. et al. Atomic-level engineering of phase change material for novel fast-switching and high-endurance PCM for storage class memory application. *2013 IEEE Int. Electron Devices Meet. (IEDM*) **30**, 6.1-6.4 (2013).

24. Tuma, T. et al. Stochastic phase-change neurons. *Nat. Nanotechnol*. **11**, 693 (2016).

25. Slesazeck, S. et al. Physical model of threshold switching in $NbO_2$ based memristors. *RSC Adv*. **5**, 102318-102322 (2015).

26. Gibson, G. A. et al. An accurate locally active memristor model for S-type negative differential resistance in $NbO_x$. *Appl. Phys. Lett.* **108**, 023505 (2016).
